# Identification of the Potential Pharmacotherapeutic Risk in Hospitalized Geriatric Patients

**DOI:** 10.7759/cureus.81485

**Published:** 2025-03-30

**Authors:** Anna Zupcan Vicena, Hajnalka Komjathy, Jana Urbankova, Tatiana Foltanova, Stanislava Kosirova

**Affiliations:** 1 Hospital Pharmacy, General Hospital AGEL Komarno, s.r.o., Komarno, SVK; 2 Department of Pharmacology and Toxicology, Faculty of Pharmacy, Comenius University Bratislava, Bratislava, SVK

**Keywords:** drug interactions, eu(7)-pim list, geriatric patients, hospitalizations, pharmacotherapeutic risk, potentially inappropriate medications

## Abstract

Introduction: Geriatric patients need carefully tailored pharmacotherapeutic approaches to minimize the risks of inappropriate treatment and hospitalizations. In Slovakia, there is a lack of studies documenting the medication use patterns in hospitalized geriatric patients. The primary outcome of our study was to identify potential pharmacotherapeutic risks in hospitalized geriatric patients.

Methods: We performed a retrospective analysis of the medical records from 122 patients (65 years and older) hospitalized in the geriatric department. Potential pharmacotherapeutic risk was assessed by identifying potential drug-drug interactions (PDIs) and potentially inappropriate medications (PIMs). PDIs were identified using the Lexicomp® database, and PIMs were evaluated using the EU(7)-PIM list. PDIs were classified into five severity categories: A (unknown), B (minor), C (moderate), D (major), and X (contraindicated). Patients’ medication was labeled as "PDIs in the therapy" or "PIMs in the therapy" based on the presence of PDIs or PIMs. If PDIs were identified, the patient was labeled "PDI in therapy"; if PIMs were found, the label "PIMs in the therapy" was applied.

Results: Polypharmacy (61.5%, 75 patients) and hyperpolypharmacy (16.4%, 20 patients) were highly prevalent. PDIs were identified in 80.3% of patients (98 patients). Out of the total 417 PDIs, eight were contraindicated (type X) and 47 were classified as major (type D) interactions. PIMs were used by 73.8% of patients (90 patients), with the most common being pantoprazole (37.7%, 46 patients), alprazolam (27.8%, 34 patients), and digoxin (15.6%, 19 patients). We found a moderate relationship between the number of used drugs and the number of PIMs (ρ = 0.574, R^2 ^= 0.8697, p < 0.001). The mean number of hospitalizations was higher in patients with PDI in therapy (p < 0.001), had a weak relationship with the number of PDIs (p < 0.05), and did not correlate with the number of used drugs or PIMs. Only 6.6% of patients (eight patients) had no potential pharmacotherapeutic risk, while 32% (39 patients) had mild risk (PDIs or PIMs), and 61.5% (75 patients) had high risk (both PDIs and PIMs in the therapy).

Conclusion: Our results underscore the urgent need for medication reviews by hospital pharmacists and the active promotion of drug deprescription among clinicians.

## Introduction

Ageing is characterized by distinct physiological and pathological complexities influencing disease progression and therapeutic interventions. Recognizing these challenges, the World Health Organization has designated 2020-2030 as the Decade of Healthy Ageing, emphasizing the urgent need for evidence-based strategies to promote well-being in older populations. The growing demographic shift toward an ageing population presents multifaceted challenges for healthcare professionals and interdisciplinary sectors, raising concerns about the sustainable management of increasing healthcare expenditures and the socioeconomic implications of a diminishing younger workforce [1]. We are witnessing a global increase in medicine use due to their increased availability [2]. Treatment regimens are typically guided by condition-specific clinical protocols in the management of polymorbid geriatric patients. This practice often requires the concurrent use of multiple medications, which increases the likelihood of polypharmacy and, in more complex cases, polypragmasia in elderly populations. The Slovak Republic faces this challenge alongside other nations as it strives to optimize care for its ageing citizens.

Health insurance companies' expenditures on prescription drugs increased from €1060.3 million in 2018 to €1232.08 million in 2022 [3]. Polypharmacy is a huge problem, and it is also one of the main factors that increase the rate of prescription of potentially inappropriate medications (PIMs) [4,5] and thus pharmacotherapeutic risk. The presence of PIMs in therapy may increase the medication-related adverse effects and contribute to an increased rate of hospitalizations and falls in geriatric patients [6]. Various tools, such as explicit criteria, are regularly updated and could be used to prevent PIM prescriptions. These criteria include Beer's criteria [7], Screening Tool of Older Persons' Prescriptions (STOPP)/Screening Tool to Alert to Right Treatment (START) criteria [8], PRISCUS list [9], or EU(7)-PIM list [10]. Besides this, prescribing medications should be individualized as much as possible, rational, safe, and simultaneously conducted with the least number of medications and the lowest cost to the patient. Therefore, pharmacotherapy of geriatric patients should always be a part of the overall therapeutic approach to the patient. It should be simple, modern, beneficial, and individual [11].

As described before, pharmacists are essential in conducting medication reviews, enhancing pharmacotherapy, and reducing adverse outcomes for geriatric patients. Their expertise allows them to review the treatment of chronic conditions effectively by detecting and preventing medication errors and drug-related issues and optimizing medication use [12].

Based on these facts, we decided to evaluate the pharmacotherapeutic risk of patients hospitalized in the geriatric ward in our hospital and identify the occurrence of polypharmacy, potential drug-drug interactions (PDIs), and PIMs. However, contrary to other countries, the role of a hospital pharmacist is very limited in the Slovak Republic. With this study, we want to draw attention to the importance of the presence of pharmacists within multidisciplinary teams.

This study aimed to systematically identify and evaluate potential pharmacotherapeutic risks in hospitalized geriatric patients in Slovakia. Specifically, the focus was on assessing the prevalence of polypharmacy, hyperpolypharmacy, PDIs, and PIMs, using established tools such as the Lexicomp® database for PDIs and the EU(7)-PIM list for PIMs.

## Materials and methods

Patient selection and data recording

The study was conducted retrospectively in the General Hospital AGEL Komarno, s.r.o., in cooperation with the hospital pharmacy and the department for geriatric patients. A hospital pharmacist collected all data retrospectively from the electronic records of patients' admissions on Mondays of every week from September to December 2018. The Ethics Committee at the General Hospital AGEL Komarno, s.r.o., approved the project and agreed to the publication of the obtained data.

The inclusion criteria included age of 65 and older, admission to the hospital on the day of follow-up, and admission at most a week ago (to prevent the analysis of the same patient's medication history twice).

Exclusion criteria were antibiotic treatment, none or one drug in therapy, discharge on the day of follow-up, multiple sclerosis, or terminal illness. Antibiotic treatment was excluded from the study to maintain a focus on chronic pharmacotherapeutic risks, such as drug-drug interactions and PIMs, which are more relevant to long-term medication use and to avoid confounding factors associated with acute infection management.

The sample size was calculated based on the total number of hospitalized geriatric patients (aged 65 and over) in the Slovak Republic for the year 2017, which was 418,764 patients [3]. Using a 95% confidence interval and a 10% margin of error, the calculated sample size was 97 patients. In order to enhance statistical power and reduce variability, the study included a larger number of participants than the calculated sample size.

A total of 127 patients hospitalized in the department met the inclusion criteria and were initially enrolled in the study. However, the final analysis included 122 patients. Five patients were excluded from the analysis because they were not receiving any pharmacological treatment. 

The recorded data were demographical (age, sex), pharmacotherapeutic (all used drugs with doses), and the number of hospitalizations in the observed period. Pharmacotherapeutic data were accessed from the hospital pharmacy software Promis® Lozis. To analyze pharmacotherapy, the brand names, strengths, and dosages of all drugs were recorded. The active substances were identified, and the daily doses of all drugs used were counted.

Polypharmacy was defined as using 5-9 medications simultaneously, while hyperpolypharmacy (excessive polypharmacy) was defined as using ten or more medications simultaneously [13].

PDI identification

Patients' pharmacotherapies were analyzed using the Lexicomp® program. Only interactions type X, D, and C were recorded for our analysis. The minor interactions (type B) and unknown interactions (type A) were excluded from our analysis because they lack clinical significance, pose minimal risk to patient safety, and do not warrant the allocation of resources compared to more serious interactions. The interactions of type X are contraindicated; therapy modification is recommended for type D, and more frequent monitoring of the patient's condition is recommended for type C. All identified interaction pairs were recorded in MS Excel (Microsoft Corporation, Redmond, Washington, United States), divided according to the type of interaction, and counted and processed by the IBM SPSS Statistics for Windows, Version 19 (Released 2010; IBM Corp., Armonk, New York, United States) as described below.

PIM identification

The EU(7)-PIM (2015) list [10] was used to identify PIMs. In the EU(7)-PIM list, some drugs are classified as PIMs only when used at doses exceeding specific thresholds, such as spironolactone (>25 mg/day), ibuprofen (>3 × 400 mg/day), haloperidol (>2 mg single dose; >5 mg/day), lorazepam (>1 mg/day), lormetazepam (>0.5 mg/day), zopiclone (>3.75 mg/day), and zolpidem (>5 mg/day). If a given drug was present at the specified dose in the patient's pharmacotherapy, we considered it potentially inappropriate. Given that the cohort consisted of hospitalized patients, the duration of drug use was deemed less relevant for this analysis, as the focus was on the immediate pharmacotherapeutic risks during the hospital stay.

Statistical analysis 

All data were statistically analyzed using IBM SPSS Statistics for Windows, Version 19 (Released 2010; IBM Corp., Armonk, New York, United States). The variables with/without PDI, with/without PIMs, polypharmacy/hyperpolypharmacy, and hospitalizations 1x/hospitalizations more than 1x were created for further analysis from the obtained primary data.

Pharmacotherapeutic risk was stratified into three distinct categories based on the presence of PDIs and PIMs in a patient’s therapy. The low-risk category included patients with no identified PDIs or PIMs in their medication regimen. The mild-risk category encompassed patients who had either PDIs or PIMs present in their therapy, but not both. Finally, the high-risk category was assigned to patients who had both PDIs and PIMs identified in their therapy, indicating a more significant potential for adverse outcomes. 

The data in the results are expressed as percentages (categorical variables) or means of the group (continuous variables) with standard error of the mean (SEM), minimal and maximal values, and group mode. The differences between groups were evaluated using Student's t-test and analysis of variance (ANOVA). The relationships between the variables were determined using Spearman's rank-order correlation and logistic regression. Spearman's correlation was used to determine the relationship between the number of drugs used and PDIs/PIMs/hospitalizations. The correlation strength was set as follows: 0.00-0.19, very weak; 0.20-0.39, weak; 0.40-0.59, moderate; 0.60-0.79, strong; and 0.80-1.0, very strong. Odds ratios (ORs) and corresponding 95% confidence intervals (CIs) were calculated from the drugs used, PDIs, and PIMs associated with the hospitalization rate. The p-value of <0.05 was considered statistically significant.

## Results

The medication use in the cohort

There were 122 patients included in our cohort. The mean age was 83.79 ± 0.45 years (69-95; 80). Women prevail over men (71.30% vs 28.70%). In total, patients took 808 drugs per day (6.62 ± 0.25 drugs/day (2-15; 5)) (Table 1). Polypharmacy was found in 61.5% of patients, and hyperpolypharmacy was found in 16.4%. The mean number of drugs used in patients with hyperpolypharmacy was significantly higher compared to patients with polypharmacy (p < 0.001) (Table 1).

**Table 1:** PDIs and PIMs identified in the studied group of patients PDIs: potential drug-drug interactions; Nb of drugs: total number of drugs per day; Mean: mean number of drugs per patient per day; SEM: standard error of the mean; N: number of patients in the group

	All patients (N = 122)		Without polypharmacy (N = 27)		Polypharmacy (N = 75)		Hyperpolypharmacy (N = 20)	
	Nb of drugs	Mean ± SEM	Nb of drugs	Mean ± SEM	Nb of drugs	Mean ± SEM	Nb of drugs	Mean ± SEM
All drugs	808	6.62 ± 0.25	86	3.19 ± 0.15	501	6.68 ± 0.16	221	11.05 ± 0.26
PDIs whole cohort	417	3.42 ± 0.29	105	3.89 ± 0.73	245	3.26 ± 0.35	67	3.35 ± 0.73
PDIs type X	8	0.07 ± 0.02	3	0.11 ± 0.06	5	0.05 ± 0.03	0	0.00 ± 0.00
PDIs type D	47	0.39 ± 0.06	13	0.48 ± 0.14	28	0.37 ± 0.08	6	0.30 ± 0.11
PDIs type C	362	2.97 ± 0.27	89	3.30 ± 0.68	212	2.83 ± 0.31	61	3.05 ± 0.70
PIMs	184	1.48 ± 0.11	11	0.41 ± 0.11	122	1.63 ± 0.14	51	2.40 ± 0.25

The identification of the PDIs

At least one PDI was identified in 80.3% (98) of patients. The mean PDIs were 3.42 ± 0.29 PDI/patient (0-13; 0). We identified 417 potential interactions in the cohort, of which eight were type X-contraindicated (Table 2). 

**Table 2:** Identified potential drug-drug interactions of type X in cohort and recommendations according to the Lexicomp® and EU(7)-PIM list s.c.: subcutaneous; LMWH: low-molecular-weight heparin

Drug A	Drug B	Recommendation
Quetiapine	Escitalopram	Reconsider the use of quetiapine, if possible, substitution with another psycholeptic drug
Quetiapine	Potassium chloride	Reconsider the use of quetiapine; potassium chloride administered in a liquid form
Diphenoxylate/atropine	Potassium chloride	Administer an antidiarrheal agent for a short time while symptoms last; potassium chloride is administered in liquid form with a time distance
Rasagiline	Cyproheptadine	Reconsider the use of cyproheptadine, discontinue if possible
Rasagiline	Trazodone	Replacement of rasagiline with another antidepressant, e.g., escitalopram
Buprenorphine	Tramadol	Use only one analgesic according to pain intensity, and follow the analgesic ladder
Enoxaparin	Clopidogrel	During the use of s.c. LMWH discontinue clopidogrel
Dabigatran etexilate	Dalteparin	When switching from s.c. LMWH to oral anticoagulants, keep the patient under surveillance, administer LMWH in the morning, anticoagulant at 12 hours late, and shift the dabigatran administration time six hours earlier on the following two days

The differences between the means of PDIs in patients with polypharmacy and hyperpolypharmacy were not statistically significant (Table 1). Patients with identified PDIs in their therapy used a significantly higher number of medications compared to those without PDIs in their treatment regimen (6.85 ± 0.29 vs. 5.71 ± 0.38, p < 0.05, Table 3).

**Table 3:** The mean number of drugs, PDIs, potentially inappropriate medications, and hospitalizations analyzed in the cohort N: number of patients; Nb: number; PDIs: potential drug-drug interactions; PIMs: potentially inappropriate medications; mean: mean number of drugs per patient per day; SEM: standard error of the mean; NS: nonsignificant For statistical analysis, independent sample T-test was performed

Variable /patients	With PDI (N = 98)	No PDI (N = 24)	t-value	p-value	With PIM (N = 91)	No PIM (N = 31)	t-value	p-value
	Mean ± SEM				Mean ± SEM			
Nb of drugs	6.85 ± 0.29	5.71 ± 0.38	2.373	p < 0.05	7.36 ± 0.27	1.96 ± 0.35	6.570	p < 0.001
Nb of PDIs	4.24 ± 0.31	0	13.689	p < 0.001	3.19 ± 0.33	3.38 ± 0.61	18.667	NS
Nb of PIMs	1.58 ± 0.13	1.08 ± 0.19	2.170	p < 0.05	1.02 ± 0.11	0	-0.580	p < 0.001
Nb of hospitalizations	2.86 ± 0.21	1.83 ± 0.14	3.999	p < 0.001	1.82 ± 0.19	2.32 ± 0.42	-0.347	NS

The use of PIMs 

A total of 73.8% of patients used at least one PIM. We identified 184 PIMs in the whole cohort. The mean number of PIMs used per patient was 1.48 ± 0.11 PIMs/day (0-5; 1).

We found a significantly higher average number of PIMs in patients with hyperpolypharmacy than in those with polypharmacy (p < 0.01, Table 1). We identified 42 different PIMs in the cohort. ATC classes N (32.1%), C (25.0%), and A (23.46%) were mainly used. The most frequently used PIMs were pantoprazole (37.78%), alprazolam (27.78%), and digoxin (15.56%). We confirmed a moderate relationship between the number of drugs taken and the number of PIMs (ρ = 0.574, R^2^ = 0.8697, p < 0.001).

The rate of hospitalizations and the pharmacotherapeutic risk 

The mean number of hospitalizations in the observed period was 2.66 ± 0.18 (1-13; 1) times/patient. A total of 31.10% of the patients were hospitalized once, 24.60% were hospitalized twice, and 23.80% were hospitalized thrice. We did not find any significant differences in the number of drugs used (p = 0.051), PDIs (p = 0.084), or PIMs (p = 0.182) in patients who were hospitalized more than once compared to those patients hospitalized only once.

There was a significantly higher rate of hospitalizations in patients with PDIs in therapy compared to patients without PDIs (with PDIs 2.86 ± 0.21 times vs no PDIs 1.83 ± 0.14 times, p < 0.001). The relationship between hospitalizations and the presence of PDIs was moderate (ρ = 0.411, p < 0.001) in patients who were hospitalized more than once.

The difference in the rate of hospitalizations between the patients with polypharmacy (2.55 ± 0.21 times) and hyperpolypharmacy (2.85 ± 0.38 times) was not statistically significant (p = 0.490), nor was it between the patients with the presence of PIMs (with PIM 2.62 ± 0.19 times vs no PIM 2.77 ± 0.42 times, ρ = 0.018, NS).

When analyzing the correlations between the observed variables and the number of hospitalizations, we found that the number of hospitalizations was neither correlated with the number of medications taken (ρ = 0.054, R^2^ = 0.0178, NS) nor with the number of PIMs (ρ = -0.002, R^2^ = 0.1249, NS) but had a weak correlation with the number of PDI (ρ = 0.19, R^2^ = 0.5086; p < 0.05)

Potential pharmacotherapeutic risk was assessed as the presence of PDIs and PIMs in therapy. Only 6.6% of patients in our cohort had no potential pharmacotherapeutic risk. In 32% of patients, we identified mild risk (PDIs or PIMs); in 61.5%, we identified high risk (both PDIs and PIMs in the therapy).

We did not find any statistically significant difference in the odds ratio (OR) of the factors that might have affected the rate of hospitalizations. However, hyperpolypharmacy was the strongest of the factors (OR: 3.20; 95% CI: 0.844-12.135; p = 0.087) (Table 4).

**Table 4:** Factors affecting the rate of hospitalizations Nb: number; PDI: potential drug-drug interaction; PIM: potentially inappropriate medications

Variables	OR	95% CI	p-value
Nb of drugs	1.152	0.956-1.389	0.138
Nb of PDIs	1.122	0.982-1.283	0.091
Nb of PIMs	1.056	0.707-1.578	0.790
Nb of PIMs	1.140	0.413-3.148	0.801
PDIs or PIMs in therapy	0.960	0.200-4.614	0.959
PDIs and PIMs in therapy	1.650	0.361-7.545	0.518
Polypharmacy	1.927	0.778-4.775	0.156
Hyperpolypharmacy	3.200	0.844-12.135	0.087

## Discussion

Polymorbidity is a characteristic feature of geriatric patients, and such patient is characterized by poor health outcomes, higher risk of falls, reduced quality of life, psychological distress, more postoperative complications, longer length of hospital stay, higher healthcare costs, and higher mortality [14]. An incorrectly administered drug, an inappropriate drug, or clinical malpractice may result in a greater safety risk. Making decisions about the appropriateness of pharmacotherapy in older people is based on age rather than individual examination of relevant factors [15]. Polypharmacy, which is often connected to polymorbidity, increases the risk of drug interactions and PIMs. That endangers geriatric patients with increased adverse drug reactions, complications, and increased rates of hospitalizations [16]. Our study evaluated the pharmacotherapeutic risk defined by the number of drugs used, their appropriateness for geriatric patients, and potential drug interactions. We investigated associations between the observed variables and possible correlations between these factors and the number of hospitalizations.

In a recent analysis, Yoshida et al. suggested that the number of drugs, especially hyperpolypharmacy, can be used as an indicator of physical frailty in geriatric patients. Thus, the treatment plan should emphasize medication management while proactively preventing the development of frailty and geriatric syndromes [17]. In this study among hospitalized geriatric patients, polypharmacy (61.5%) and hyperpolypharmacy (16.4%) were presented extensively, similar to our previous study in nursing homes (polypharmacy in 83% of patients) [18] and ambulatory settings (polypharmacy in 69%) [4]. The positive is that since 2016, when the first study was conducted on hospitalized patients in our hospital, the polypharmacy has not increased extensively [19]. However, the hyperpolypharmacy was not evaluated back then. Since polypharmacy and hyperpolypharmacy are problems in other countries as well [20], Yoshida et al. suggest implementing effective regulatory and policy measures and integrating geriatrician and clinical pharmacy services to prevent increased safety risks for patients [21]. In our study, hyperpolypharmacy increased the risk of hospitalizations 3.20 times. However, it was not statistically significant (p = 0.087). Besides this, we identified at least one potential interaction in 80.3% of our patients. The total number of identified interactions was 417. However, the clinical manifestation of potential interaction strongly depends on individual patients. The identified interactions were considered potential and thus may not have manifested in the patient, but their manifestation was not excluded. In our study, we consulted all eight found contraindicated interactions (type X) with the attending physician. Only in one case was the use of two contraindicated drugs justified. That was the combination of dabigatran and dalteparin, which was deployed due to the transition of the patient from the intramuscular treatment to the peroral drug form. Based on our recommendations, the attending physician changed the patients' therapy in seven cases. All recommendations about identified contraindicated PDIs according to the Lexicomp® and EU(7)-PIM list are displayed in Table 2.

Besides PDI, as the factor that increases the potential pharmacotherapeutic risk, we assessed the use of PIMs as well. Experts in countries around the world are constantly trying to create and revise lists of drugs that could facilitate decision-making about the appropriateness of the use of particular drugs in patients [7,8,10]. We used the EU7-(PIM) list [10] in our study. The use of at least one PIM was identified in 73.8% of patients, which is higher than other studies [22] but similar to our earlier study among geriatric patients in ambulatory care [4]. The most frequently used PIM among our patients was pantoprazole (37.78%), similar to our previous study in ambulatory settings [4]. Proton pump inhibitors (PPIs) belong to the most prescribed drugs in the world, and in recent decades, their prescription has increased continuously. As they are prescribed to patients with gastrointestinal diseases, during hospitalization, and when taking gastrotoxic drugs, geriatric patients are most often treated with them. To avoid potential adverse effects caused by their overuse, the FDA has published a list of approved indications for the treatment with PPIs that describes the length of treatment and the dose of the appropriate drug [23]. However, inappropriate prescribing of PPIs still occurs, primarily due to physicians repeatedly prescribing them without reassessing the risk/benefit ratio of long-term treatment, or their use is not discontinued after discharge from the hospital [24]. In patients using PPIs due to unclear underlying indications, a gradual withdrawal of the drug is necessary. As sudden cessation may cause gastric acid hypersecretion, it is essential to gradually decrease doses, use intermittent regimens, or replace PPIs with other acid-reducing agents such as H2-receptor blockers [25]. It is known that the higher the number of medications a patient takes, the greater the likelihood of drug-drug interactions, PIM incidence, and adverse drug reactions [18]. We also observed this in our study when we found a moderate relationship between the number of drugs taken and the number of PIMs. Similarly, patients with PDIs used a significantly higher number of drugs and had a significantly higher number of PIMs compared to patients without PDIs in therapy (p < 0.05).

However, inappropriate prescribing is common in geriatric patients; it is connected to adverse drug reactions, increased rate of hospitalizations, functional impairment, and inefficient use of financial resources [26]. During our four-month data collection period, the average hospitalization rate was 2.66 times/patient and was higher in patients with PDI in therapy (p < 0.001). Another important finding was the moderate correlation between the number of hospitalizations and the presence of PDIs in patients who were hospitalized more than once. Contrary to our expectations, no correlation was found between the number of drugs taken or PIMs and the rate of hospital admissions. However, we assessed a weak relationship with the number of PDIs (p < 0.05). Medication-related hospital admissions could be prevented by early assessment of the medication regimen and, with such intervention, significantly and substantially reduce the risk of adverse drug reactions [27]. Due to the insufficient information on the pharmacotherapy of geriatric patients from randomized controlled trials, a specific approach for these patients is necessary. At the same time, it is essential to respect the changes in the pharmacokinetics and pharmacodynamics of drugs because, in geriatric patients, there is a higher incidence of specific drug reactions [28]. While evaluating the potential pharmacotherapeutic risk in our study, we found that only 6.6% of patients without any risk. In 32% of patients, we identified mild risk, and in 61.5% of patients, we identified high potential pharmacotherapeutic risk. A recent study from Canada identified polypharmacy, hyperpolypharmacy, and PIM use as the factors associated with three-year mortality and institutionalization [29] in geriatric patients. Although, in our study, we did not assess the risk of mortality, our results may contribute to raising awareness of proper pharmacotherapy for geriatric patients. To optimize the pharmacotherapy of geriatric patients, health professionals should be more aware of inappropriate drug lists, and healthcare must be a public policy priority. The high potential for deprescribing enhances patient safety and reduces polypharmacy. A pharmacist's geriatric assessment and deprescribing-focused medication review represent a personalized approach needed in treating geriatric patients to increase the safety of their pharmacotherapy [30]. If pharmacists are skilled, they can advise clinicians in multidisciplinary teams to improve health outcomes, quality of life, and cost-effectiveness in treating geriatric patients [12].

Strengths and limitations of the study

The study is a retrospective analysis, which relies on the accuracy and completeness of medical record data. PDIs were identified using the Lexicomp® database, which provides a standardized and widely recognized tool for PDI assessment. The limitations of our study include a small patient sample size determined by the number of admissions to the ward during the specified period. The small sample size may limit the generalizability of the findings to the broader population of hospitalized geriatric patients in Slovakia. The clinical manifestation of potential interactions depends heavily on the individual patient; while the identified interactions were potential and may not have manifested, their occurrence could not be excluded. Similarly, we did not follow the justification of PIM use in patients' therapy, nor the duration of the PIM use, since it was a hospital setting. 

## Conclusions

This study highlights significant pharmacotherapeutic risks among hospitalized geriatric patients in Slovakia, with polypharmacy and hyperpolypharmacy being highly prevalent. A substantial proportion of patients were found to have PDIs, including contraindicated and major interactions, while the use of PIMs was also widespread. Patients with PDIs experienced significantly more hospitalizations, and there was a notable association between PDIs and increased hospitalization frequency. Alarmingly, the majority of patients faced high pharmacotherapeutic risk due to the co-occurrence of PDIs and PIMs, while only a small fraction had no identifiable risk factors, underscoring systemic challenges in medication safety.

These findings emphasize the critical need for structured interventions, including routine medication reviews by hospital pharmacists and deprescribing initiatives targeting high-risk medications like PPIs, benzodiazepines, and cardiac glycosides. The strong association between PDIs and hospitalizations suggests that optimizing drug regimens could reduce healthcare utilization. Given Slovakia’s documented gaps in geriatric prescribing guidelines and limited specialist availability, implementing standardized protocols aligned with EU7-PIM criteria and enhancing interdisciplinary collaboration are urgent priorities to mitigate risks in this vulnerable population.
